# Apelin/APJ relieve diabetic cardiomyopathy by reducing microvascular dysfunction

**DOI:** 10.1530/JOE-20-0398

**Published:** 2021-01-25

**Authors:** Bin Li, Jiming Yin, Jing Chang, Jia Zhang, Yangjia Wang, Haixia Huang, Wei Wang, Xiangjun Zeng

**Affiliations:** 1 School of Basic Medical Sciences, Capital Medical University, Beijing, China; 2 Beijing You An Hospital, Capital Medical University, Beijing, China; 3 Beijing Institute of Hepatology, Beijing, China; 4 Beijing Lab for Cardiovascular Precision Medicine, Beijing, China

**Keywords:** diabetic cardiomyopathy, microcirculatory injuries, endothelial dysfunction, apelin, APJ

## Abstract

Microcirculatory injuries had been reported to be involved in diabetic cardiomyopathy, which was mainly related to endothelial cell dysfunction. Apelin, an adipokine that is upregulated in diabetes mellitus, was reported to improve endothelial cell dysfunction and attenuate cardiac insufficiency induced by ischemia and reperfusion. Therefore, it is hypothesized that apelin might be involved in alleviating endothelial cell dysfunction and followed cardiomyopathy in diabetes mellitus. The results showed that apelin improved endothelial cell dysfunction via decreasing apoptosis and expression of adhesion molecules and increasing proliferation, angiogenesis, and expression of E-cadherin, VEGFR 2 and Tie-2 in endothelial cells, which resulted in the attenuation of the capillary permeability in cardiac tissues and following diabetic cardiomyopathy. Meanwhile, the results from endothelial cell-specific APJ knockout mice and cultured endothelial cells confirmed that the effects of apelin on endothelial cells were dependent on APJ and the downstream NFκB pathways. In conclusion, apelin might reduce microvascular dysfunction induced by diabetes mellitus via improving endothelial dysfunction dependent on APJ activated NFκB pathways.

## Introduction

Diabetic cardiomyopathy is known to be a special form of heart disease, first proposed by Rubler *et al.* (1972), and its typical definition includes abnormal structural and functional abnormalities in the myocardium of diabetic patients without coronary artery disease and/or hypertension (Rubler *et al.* 1972). In the early stage of diabetic cardiomyopathy, metabolic disorders lead to diastolic dysfunction in diabetic patients (Adeghate & Singh 2014, Jia *et al.* 2016), and in the late stage of diabetic cardiomyopathy, the structural changes in the heart causes systolic dysfunction and heart failure (Aneja *et al.* 2008). Besides dysfunction of cardiomyocytes, microvascular dysfunction is critical in inducing diabetic cardiomyopathy (Wang *et al.* 2016). Studies have revealed that diabetes impairs the function and structure of myocardial microvascular vessels both in diabetic patients and diabetic animal models (Aneja *et al.* 2008, Campbell *et al.* 2011), of which impaired endothelial cells were considered as the origin of microvascular dysfunction in early type 2 diabetes (Hahad *et al.* 2019). Therefore, how did the myocardial endothelial cells were impaired in diabetes were important to understand and prevent diabetic cardiomyopathy (Hahad *et al.* 2019).

It is, generally, believed that hyperglycemia was the primary factor causing endothelial dysfunction in diabetic cardiomyopathy (Jia *et al.* 2018). However, microvascular dysfunction or diabetic cardiac complications were also observed in diabetic patients whose blood glucose was controlled in the normal range, which were defined as a phenomenon ‘hyperglycemic memory’ (Lee *et al.* 2019). Therefore, it was supposed that there must be other factors causing endothelial injuries and resulted in microvascular dysfunction besides high blood glucose in diabetic patients.

Apelin, an adipokine, was observed increasing in diabetic patients and animal models (Boucher *et al.* 2005). And studies have implicated that apelin induced proliferation (Kasai *et al.* 2004), angiogenesis (Kasai *et al.* 2010), endothelial to mesenchymal transformation (Yu *et al.* 2019) and decreased permeability of endothelial cells (Zhang *et al.* 2014) via combing with its receptor APJ. These raised a possibility that apelin might be related to the endothelial injuries and microvascular dysfunction in diabetic cardiomyopathy. In this study, the role of apelin and its receptor APJ in endothelial dysfunction induced by diabetes mellitus and following diabetic cardiomyopathy were investigated in diabetic animal models.

## Materials and methods

### Ethical statement

All animal studies followed the Animal Care and Use Committee of Capital Medical University (AEEI-2016-055). All animals received humane care, and the experimental protocol was approved by the Committee of Laboratory Animals according to the institutional guidelines.

### Animal models

kkAy mice were used as a polygenic model for human type 2 diabetes mellitus (Zhang *et al.* 2013) due to the obesity, hyperglycemia, severe insulin resistance, dyslipidemia and hypertension displayed in the mice. Type 1 diabetic model was repeated by injecting intraperitoneally with STZ (40 mg/kg/day) for 5 consecutive days to APJ^fl/fl^ or APJ^ΔEC^ mice at 8 weeks of age. And the model was considered as successful when the random blood sugar exceeded 250 mg/dL (13.9 mmol/L) as reported earlier (Manoylov *et al.* 2019).

Male kkAy mice and control C57BL/6 mice at the age of 8 weeks were purchased from Capital Medical University (Beijing, China). They were housed in air-conditioned, specific pathogen-free animal quarters with lighting from 08:00  h to 21:00 h, and the mice were given unrestricted access to water throughout the study. Meanwhile kkAy mice were fed on semi-purified moderately high-fat diet containing 24% kcal fat and 0.2% cholesterol and C57/BL mice were given standard laboratory chow.

The mice were randomly divided into saline group (C57+saline group, *n* = 6, and kkAy+saline group, *n* = 6), which were intraperitoneally infused (using micro-osmotic pump form alzet, MODEL 1004, DURECT Corporation, Cupertino, CA, USA) with vehicle for 4 weeks; apelin treatment group (C57+apelin group, *n* = 6, and kkAy+apelin group, *n* = 6), which were intraperitoneally infused (using micro-osmotic pump formalzet, MODEL 1004, DURECT Corporation) with apelin-13 (A6469; Sigma-Aldrich, 30 μg/kg/day (Zhang *et al.* 2018)) for 4 weeks and F13A treatment group (C57+F13A group, *n* = 6, kkAy+F13A group, *n* = 6), which were intraperitoneally infused using micro-osmotic pump (alzet, MODEL 1004, DURECT Corporation) with F13A (the antagonist of apelin-13,057–29; Phoenix Pharmaceuticals, Strasbourg France, 25 μg/kg/day (Zhang *et al.* 2018)) for 4 weeks. And all mice were injected intraperitoneally with 5-Bromo-2’-Deoxyuridine (BrdU, 50 mg/kg/day) for 4 weeks.

APJ^fl/fl^ mice (which were acquired from KOMP) and Tie 2-Cre mice (which were as a gift from Aiquan Qu, Capital Medical University) were cross mated to get a Cre positive APJ^fl/fl^ mice (APJ^ΔEC^ mice). APJ^fl/fl^ and APJ^ΔEC^ mice were received humane care, and the experimental protocol was approved by the Committee of Laboratory animals according to the institutional guidelines.

APJ^fl/fl^ and APJ^ΔEC^ mice at 8 weeks of age were randomly divided into saline group (APJ^fl/fl^+saline group, *n* = 10, and APJ^ΔEC^+saline group, *n* = 10), which were injected intraperitoneally with saline for 5 consecutive days; STZ treatment group (APJ^fl/fl^+STZ group, *n* = 6, and APJ^ΔEC^+STZ group, *n* = 6) which were injected intraperitoneally with STZ (40 mg/kg/ day) for 5 consecutive days; STZ+apelin treatment group (APJ^fl/fl^+STZ+apelin group, *n* = 6, and APJ^ΔEC^+STZ+apelin group, *n* = 6) which were injected intraperitoneally with STZ (40 mg/kg/ day) for 5 consecutive days followed intraperitoneally infusing(using micro-osmoticpump formalzet, MODEL 1004, DURECT Corporation) with apelin-13 (A6469; Sigma-Aldrich, 30 μg/kg/day) for 4 weeks.

### Detection of heart function

Cardiac function of all animal models were measured using the Vevo 2100 imaging system (Visual Sonics Inc., Toronto, Ontario, Canada). Mice were anesthetized using tribromoethanol (100 mg/kg, Sigma-Aldrich). Ejection fraction (EF), fractional shortening (FS), cardiac output (CO) and stroke volume (SV) were calculated at M-mode to evaluate cardiac function.

### HE staining

Heart slices of all animal models were fixed with 10% formalin and then sectioned at the coronal plane. Briefly, after deparaffinization and rehydration, 4 μm longitudinal sections were stained with hematoxylin solution for 5 min followed by 5 dips in 1% acid ethanol (1% HCl in 70% ethanol) and then rinsed in distilled water. Then the sections were stained with eosin solution for 3 min and followed by dehydration with graded alcohol and clearing in xylene. The stained slices were scanned with a digital slide scanner (Pannoramic SCAN, 3DHISTECH, Budapest, Hungary).

### Masson staining

Heart slices of all animal models were fixed with 10% formalin and then sectioned at the coronal plane. The sections were deparaffinized and refixed in preheated Bouin’s Solution at 56°C for 15 min. After removing the yellow color from sections with running tap water, these sections were stained in BiebrichScarlet-Acid Fucshin for 5 min and in Phosphotungstic/Phosphomolydic Acid Solution for 5 min. Then the slices were stained with Aniline Blue Solution for 5 min to show the nucleus and mounted with xylene. The stained slices were scanned with a digital slide scanner (Pannoramic SCAN, 3DHISTECH and the blue-colored area were quantified with ImageJ software to analyze the area of fibrosis.

### PicroSirius Red (PSR) staining

Heart slices of all animal models were first immersed in Bouin’s fixative for 30 min and then washed with tap water, then the slices were immersed in Sirius Red solution (Direct Red 80 and saturated picric acid, Sigma) and briefly washed in 0.5% acetic acid (Thermo Fisher Scientific). Finally the slices were mounted with a xylene-based mounting media (Richard-Allen Scientific, Kalamazoo, MI, USA). The photos were scanned with a digital slide scanner (Pannoramic SCAN, 3DHISTECH).

### Immunostaining

Tissue sections at 4μm in each animal group were used to perform immunostaining. Incubated with the primary antibody at 4°C and then with a horseradish peroxidase-conjugated secondary antibody. Color was developed by incubating with diaminobenzidine (DAB Detection Kit, GK600505; Gnen Tech, Shanghai, China). CD31 and BrdU double staining was performed using VECTASTAIN ABC-AP Kit, alkaline phosphatase (Mouse IgG) (AK-5002, Vector Labs, Peterborough, UK) and Vector®Red (SK-5100, Vector Labs) following the manufacturer’s instructions. Hematoxylin was then used to stain the nucleus. The photos were scanned with a digital slide scanner (Pannoramic SCAN, 3DHISTECH).

### Cell culture

Native cardiac microvascular endothelial cells (NCMECs) were isolated from heart of adult male C57BL/6 mice. Briefly, mice hearts were excised and placed into the cooled PBS under sterile conditions. Then the atrium and right ventricle were removed and the left ventricle was cut into small pieces (1 mm^3^). And the cells were isolated from the left ventricle of mice by enzymatic digestion (collagenase type I, 1 mg/mL, C0130; Sigma-Aldrich; collagenase type II, 0.5 mg/mL, C6885; Sigma-Aldrich; trypsin 0.125%, C0201; Beyotime, Shanghai, China) for 2 min at 37°C in a shaking bath (repeated the operation until most of the cells had been released from tissues). Then DMEM supplemented with 10% fetal bovine serum (FBS) and 1% penicillin/streptomycin was used to terminate digestion and the whole dissociated cells were filtered through a 200-mesh sieve, centrifuged (200 ***g*** for 10 min), and then placed in the incubator at 37°C and 5% CO_2_. After 1 hrof differential adhesion, the supernatant was retained for centrifugation (700 ***g*** for 5 min). Subsequently, the cell suspensions were seeded in endothelial cell medium (ECM, 1001; ScienCell, San Diego, CA, USA) supplemented with 10% FBS, and 1% penicillin/streptomycin. The NCMECs were identified with CD31 staining, which confirmed that more than 90% of the cultured cells were CD31 positive cells. When NCMECs were well-differentiated (about 3–5 generations), they were serum-starved overnight with serum-free ECM, and then the cells were treated with HG (25 mmol/L d-glucose) and/or apelin (1.0 nmol/L) for 24 h (Yin *et al.* 2018).

### Tube formation

Using the Matrigel Basement Membrance Matrix (354234; Becton Dickinson) as the cytoskeleton, NCMECs were given different treatment (sham group; HG group: 25 mmol/L d-glucose; HG+apelin group: 25 mmol/L d-glucose+1.0 nmol/L apelin). The formation of 2D tube-like structure was observed under TH4-200 Inverted Fluorescencet Microscope (Olympus) after 24 h of treatment. The length of the tube-like structure were measured and analyzed with ImageJ software (NIH).

### Migration assay

The migration of NCMECs were assessed using a wound healing method. In brief, the cells were scratched with a tips (for pipette of 1 mL) in the middle of the dish under different conditions (Control group; HG group: 25 mmol/L D-glucose; HG+apelin group: 25 mmol/L d-glucose + 1.0 nmol/L apelin) to observe the migrated areas at different time points (0, 12, 24 h). The results were observed under TH4-200 Inverted Fluorescent Microscope (Olympus) and analysis with ImageJ software.

### Proliferation of endothelial cells

The proliferation of NCMECs was assessed* in vitro* using BrdU incorporation assay and Cell Counting Kit-8 (CCK-8, CK04; Dojindo Laboratories, Kyushu, Japan) assay, respectively.

The BrdU incorporation was detected by immunostainning with sheep anti-BrdU antibody after the cells were cultured in BrdU solution (10 μmol/L) for 24 h. Photographs were obtained using an Olympus fluorescence microscope (BX63, Olympus America Inc., Center Valley, PA, USA) at 20× magnification.

The CCK-8 test was performed following the protocol: after treatment with different conditions for 24 h, endothelial cells were treated with CCK-8 (10 µL CCK-8 reagent in 100 µL ECM) for 4 h, then the optical density (OD) was measured at 450 nm using a microplate reader (130109E; BioTek Instruments Inc., Winooski, VT, USA).

### TUNEL staining

NCMECs were fixed in 10% neutral buffered formalin; then washed with PBS and treated with 0.2% Triton X-100 for 10 min; then TUNEL reaction mixtures were added and incubated for 1 h at 37°C after blocked with normal horse serum for 30 min. Cell nuclei were stained with DAPI (ZLI-9557; ZSGB-BIO, Beijing, China). Photographs were obtained using an Olympus BX63 fluorescence microscope (Olympus America Inc., Center Valley, PA, USA) at 10× magnification. The number of TUNEL-positive cells and total cells were counted in each field using ImageJ software. The percentage of TUNEL-positive cells to total cells was calculated.

### Western blot analysis

The proteins from NCMECs were fractionated by electrophoresis on 10% SDS-PAGE, and incubated with the primary antibody followed with a horseradish peroxidase-conjugated secondary antibody. The experiment was repeated three times. The antibody to glyceraldehyde 3-phosphate dehydrogenase (GAPDH) was used to verify equal loading of proteins. Densitometry was performed with ImageJ software.

### Antibodies and reagents

Primary antibodies were rabbit anti-pIκB (2895S, Cell Signaling Technology), rabbit anti-NFκB (8242, Cell Signaling Technology), rabbit anti-pNFκB (3033, Cell Signaling Technology), rabbit anti-GAPDH (5174, Cell Signaling Technology), sheep anti-BrdU (ab1893, Abcam), mouse anti-CD31 (ab9498, Abcam), rabbit anti-CD68 antibody (ab125212, Abcam), rabbit anti-VCAM-1 (ab134047, Abcam), rabbit anti-VEGFR2 (ab 45010, Abcam), mouse anti-E-Cadherin (ab 76055, Abcam), mouse anti-ICAM-1 (ab 171123, Abcam), rabbit anti-Tie-2 (sc-9026, Santa Cruz Biotechnology) and Goat anti-mGalectin-3 (AF1197, R&D Systems). Secondary antibodies were donkey anti-mouse IgG (sc-2314, Santa Cruz Biotechnology), donkey anti-rabbit IgG-HRP (7074, Cell Signaling Technology), donkey anti-sheep IgG-555 (A21436; Invitrogen) and rabbit anti-sheep IgG (6150-04, Southern Biotech).

### Statistical analysis

Data are summarized as mean ± s.d. A value of *P* < 0.05 was considered significant. All reported significance values are two-tailed. Analyses were performed with SPSS 24 for the PC (IBM). Differences were evaluated for significance with independent Student’s *t* test (between two groups), one-way ANOVA or two-way ANOVA (used in other figures). If there are differences between the means of the different treatment groups, the significance between pair of groups would be tested using Tukey HSD method (between more than two groups).

## Results

### Apelin decreased the random blood glucose concentration in type 2 diabetic mice

Compared to C57 mice, the random blood glucose in kkAy mice were significantly increased (21.63 ± 1.03 vs 11.15 ± 0.24 mmol/L, *P* < 0.05), and apelin infusion decreased the blood glucose to 17.96 ± 0.89 mmol/L in kkAy mice, F13A increased the blood glucose to 24.26 ± 2.34 mmol/L in kkAy mice (*n* = 6; *P* < 0.05, Supplementary Fig. 2, see section on supplementary materials given at the end of this article).

### Apelin relived cardiac dysfunction in diabetic mice

**Figure 1:**
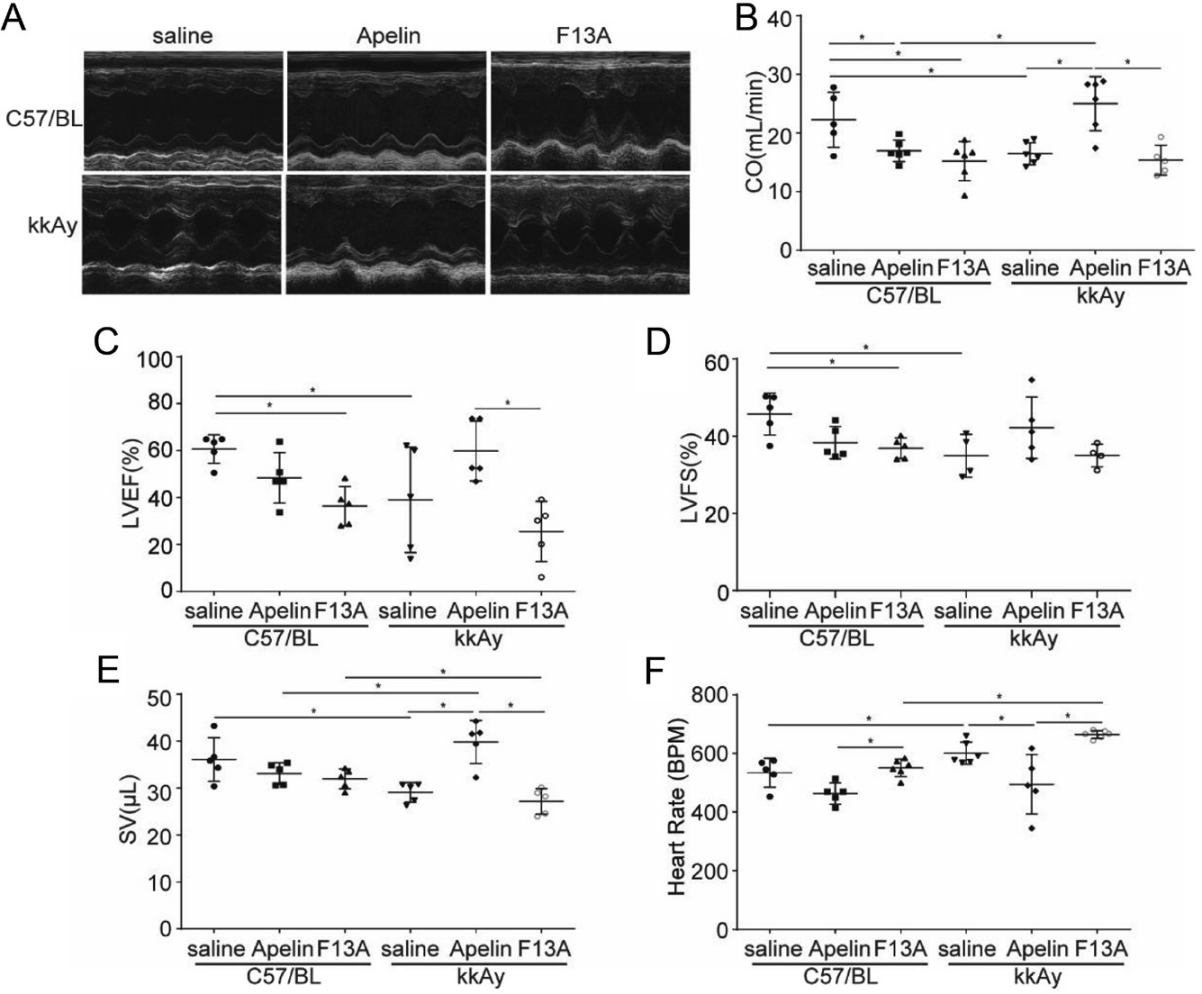
Effects of apelin on cardiac function. (A) The M-mode of echocardiography for each group. (B) Quantification of the cardiac output (CO: diabetes (*P* = 0.462), treatment (*P* = 0.051), interaction (*P* = 0.018)). (C) Quantification of the left ventricular ejection fraction (LVEF: diabetes (*P* = 0.161), treatment (*P* = 0.001), interaction (*P* = 0.031)). (D) Quantification of the left ventricular fractional shortening (LVFS: diabetes (*P* = 0.148), treatment (*P* = 0.141), interaction (*P* = 0.019)). (E) Quantification of the stroke volume (SV: diabetes (*P* = 0.020), treatment (*P* = 0.053), interaction (*P* = 0.022)). (F) Quantification of heart rate (diabetes (*P* = 0.000), treatment (*P* = 0.000), interaction (*P* = 0.176)). *n* = 6 mice per group, *P < 0.05.

To investigate the effects of apelin on cardiac function in diabetic mice, apelin and F13A were infused to the mice for 4 weeks. The results showed that at 12 weeks of age, the cardiac function of kkAy mice (SV: 29.09 ± 2.07 μL, CO: 16.47 ± 1.87 mL/min, LVEF: 69.50 ± 5.03%, LVFS: 34.93 ± 2.77%) was significantly lower than that of C57 mice (SV: 36.09 ± 6.07 μL, CO: 22.25 ± 4.68 mL/min, LVEF: 80.33 ± 1.35%, LVFS: 45.73 ± 2.41%, diabetic effects, *P* < 0.05). The 4-week treatment with apelin significantly improved the cardiac function of kkAy mice (SV: 39.85 ± 4.56 μL, CO: 25.00 ± 4.61 mL/min, HR: 601.14 ± 15.36) (*n* = 6; *P* < 0.05, Fig. 1), but without such effects in C57 mice. (Significant interaction effects between apelin and diabetic conditions, *P* < 0.05 for all.)

### Apelin decreased cardiac hypertrophy and fibrosis in diabetic mice

**Figure 2:**
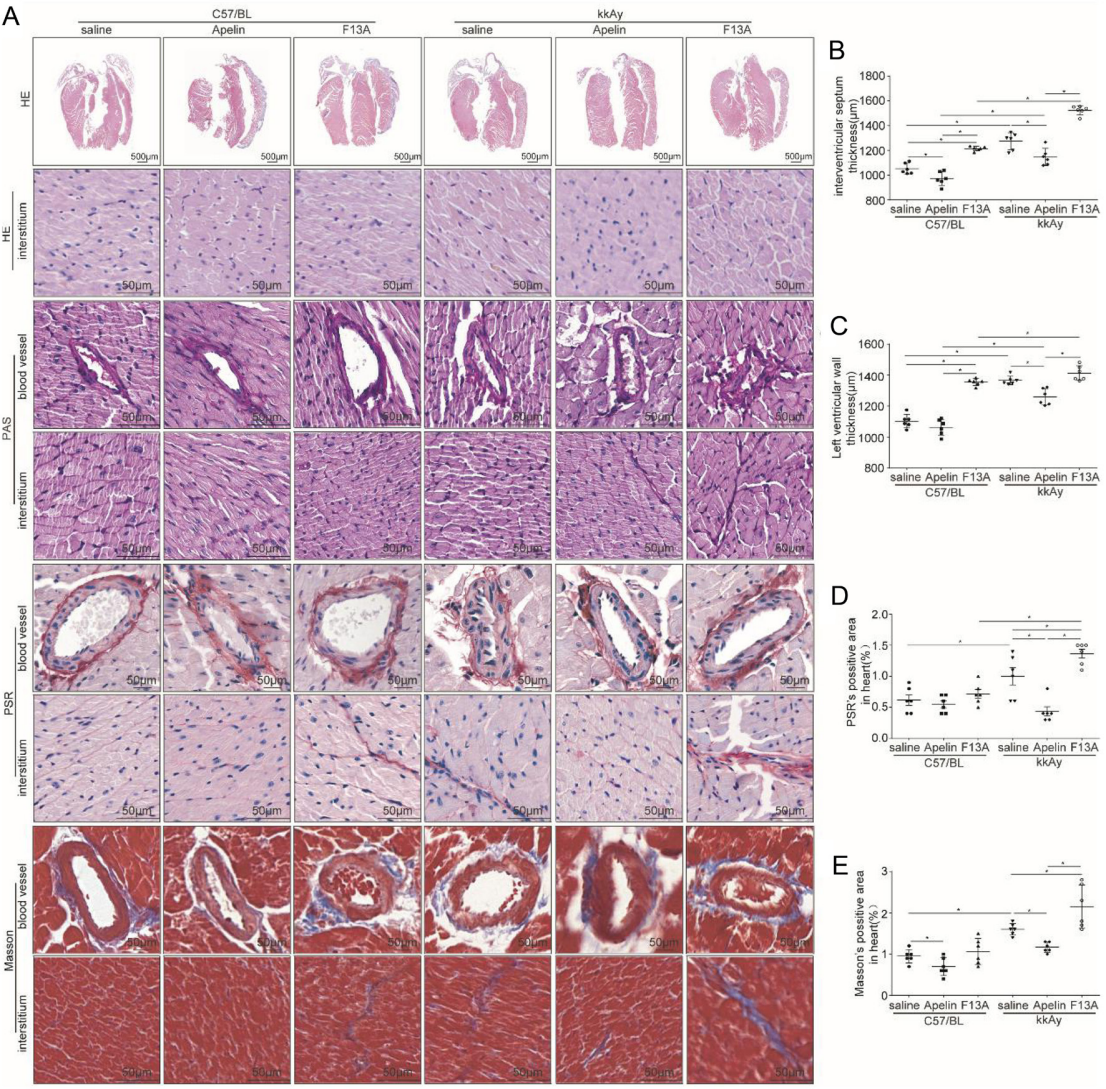
Apelin decreased cardiac hypertrophy and fibrosis in diabetic mice. (A) Representative H&E histology, periodic acid Schiff (PAS) staining, Masson Trichrome and Pico Sirius Red (PSR) staining in heart sections from C57 and kkAy mice with or without apelin/F13A treatment as quantified in (B, C, D and E) (interventricular septum thickness: diabetes (*P* = 0.000), treatment (*P* = 0.000), interaction (*P* = 0.009); left ventricular wall thickness: diabetes (*P* = 0.000), treatment (*P* = 0.000), interaction (*P* = 0.000); Masson staining: diabetes (*P* = 0.000), treatment (*P* = 0.000), interaction (*P* = 0.036); PSR staining: diabetes (*P* = 0.000), treatment (*P* = 0.000), interaction (*P* = 0.000); *n* = 6 mice per group, *P < 0.05). Scale bars represent 500 and 50 μm.

HE staining showed that the ventricular wall of kkAy mice (interventricular septum thickness: 1274.11 ± 27.76 μm; left ventricular wall thickness: 1365.43 ± 11.33 μm) were significantly thicker compared to that of C57 mice (interventricular septum thickness: 1053.05 ± 17.53 μm; left ventricular wall thickness: 1103.25 ± 17.84 μm) (diabetic effects, *P* < 0.05), which was alleviated by apelin(interventricular septum thickness: 1147.20 ± 28.08 μm; left ventricular wall thickness: 1259.31 ± 21.61 μm) in kkAy mice (significant interaction between apelin and diabetic conditions, *P* < 0.05; *n* = 6; *P* < 0.05, Fig. 2A, B and  C).

PAS staining showed that matrix deposition was significantly increased in myocardial tissue and blood vessels in kkAy mice compared to that of C57 mice (diabetic effects, *P* < 0.05), which was alleviated by apelin and aggravated by F13A (no significant interaction between apelin/F13A and diabetic conditions, *P* > 0.05, Fig. 2A).

PSR staining showed that the amount of red collagen fibers in myocardial tissue (1.00 ± 0.13%) of kkAy mice was significantly increased compared to that of C57 mice (0.61 ± 0.08%, diabetic effects, *P* < 0.01), which could be alleviated by apelin (0.43 ± 0.07%) and aggravated by F13A (1.31 ± 0.07%) in kkAy mice (significant interaction between apelin/F13A and diabetic conditions, *P* < 0.01, *n* = 6; *P* < 0.05, Fig. 2A and  D).

Masson staining showed that blue-stained collagen fibers increased in myocardial tissue of kkAy mice (1.78 ± 0.24%) compared to that of C57 mice (1.55 ± 0.31%) (diabetic effects, *P* < 0.01), which was decreased by apelin (1.18 ± 0.04%) and aggravated by F13A (2.15 ± 0.21%) in kkAy mice (significant interaction between apelin/F13A and diabetic conditions, *P* < 0.01, *n* = 6; *P* < 0.05, Fig. 2A and E).

### Apelin relived early inflammatory response in hearts of diabetic mice

**Figure 3:**
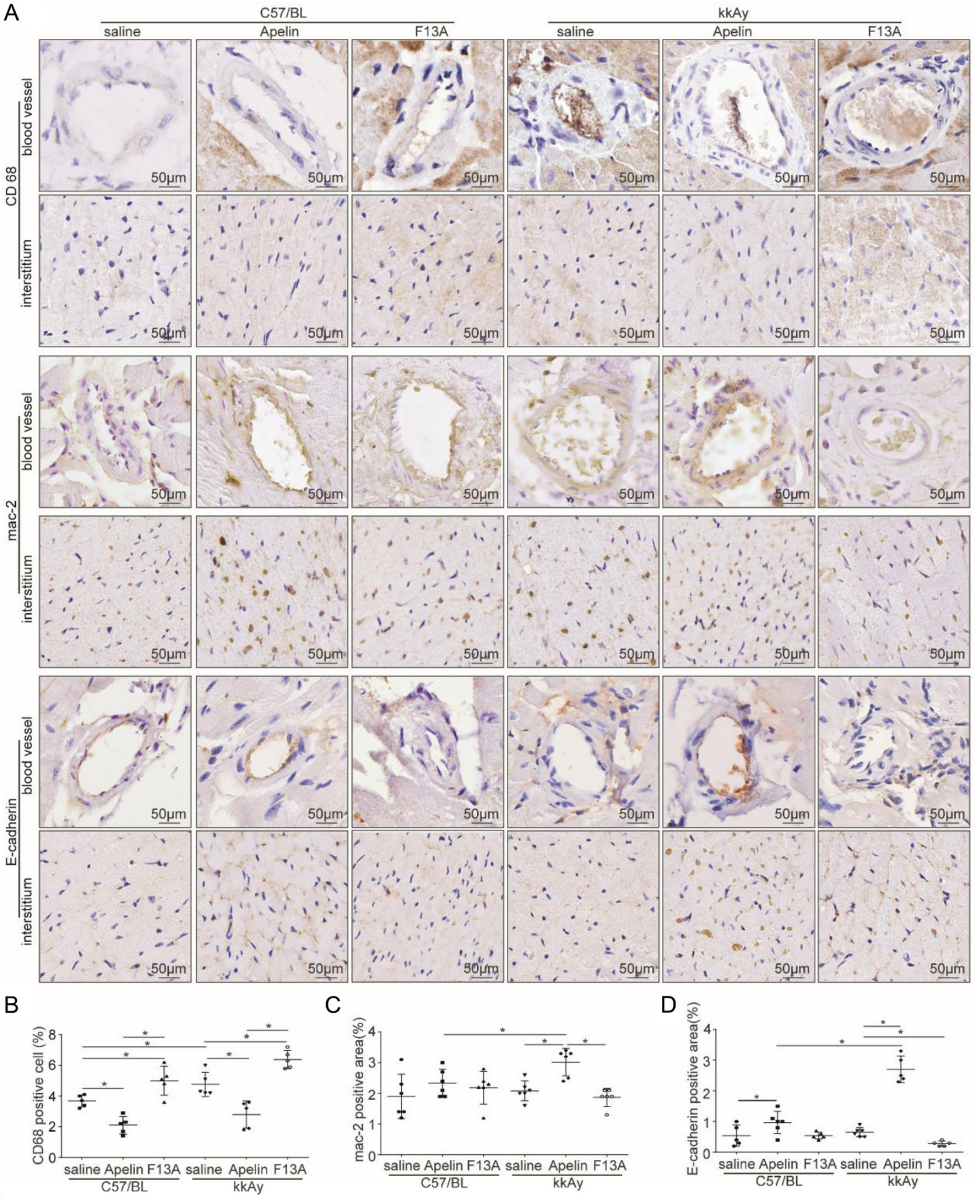
Apelin relived early inflammatory response in heart of diabetic mice. (A) Representative images of immunohistochemistry for CD68, mac-2 and E-cadherin in heart sections from C57 and diabetic mice with or without apelin/F13A treatment as quantified in (B, C and D) (CD68: diabetes (*P* = 0.001), treatment (*P* = 0.000), interaction (*P* = 0.578); mac-2: diabetes (*P* = 0.263), treatment (*P* = 0.002), interaction (*P* = 0.054); E-cadherin diabetes (*P* = 0.000), treatment (*P* = 0.000), interaction (*P* = 0.000); *n* = 6 mice per group, *P < 0.05). Scale bars represent 50 μm.

Studies have shown that inflammatory response was significantly increased in diabetic models (Lontchi-Yimagou *et al.* 2013, Mack 2018) and the early inflammatory response is mainly manifested by macrophage infiltration (Chow *et al.* 2004, Singh *et al.* 2015). Therefore, macrophages and neutrophils were detected with immunostaining in cardiac tissues. The results showed that CD68+ cells were abundantly present in myocardial tissue of kkAy mice (4.76 ± 0.35%) compared to that in C57 mice (4.12 ± 0.21%) (diabetic effects, *P* < 0.01), which was reduced by apelin (2.62 ± 0.45%) and increased by F13A (7.05 ± 0.93%) both in C57 and kkAy mice (no significant interaction between apelin/F13A and diabetic conditions, *P* > 0.05, *n* = 6; *P* < 0.05, Fig. 3A and B).

The junctions between endothelial cells have been reported to contribute to the permeability of blood vessels and induced the inflammatory reactions in tissues (Wu *et al.* 2015). The E-cadherin, one of endothelial cell-specific proteins, was detected. The results showed that the number of E-cadherin+ cells in the endothelium of arterioles of heart were significantly decreased in kkAy mice compared to C57 mice (diabetic effects, *P* <0.01), which were significantly increased by apelin (2.60 ± 0.33%) and further decreased by F13A (0.60 ± 0.14%) in kkAy mice (significant interaction between apelin/F13A and diabetic conditions, *P* < 0.01.*n* = 6; *P* < 0.05, Fig. 3A and  D). In addition, contrary to the results of CD68 staining, the results from mac-2 staining showed that mac2+ positive cells were decreased in kkAy mice compared to that of C57 mice (diabetic effects, *P* <0.01), which were significantly increased by apelin (from 2.08 ± 0.32% to 3.01 ± 0.44%) in myocardial tissue of kkAy mice (no significant interaction between apelin and diabetic conditions, *P* < 0.01. *n* = 6; *P*  > 0.05, Fig. 3A and  C).

### Apelin increased angiogenesis and proliferation of endothelial cells in heart of diabetic mice

**Figure 4:**
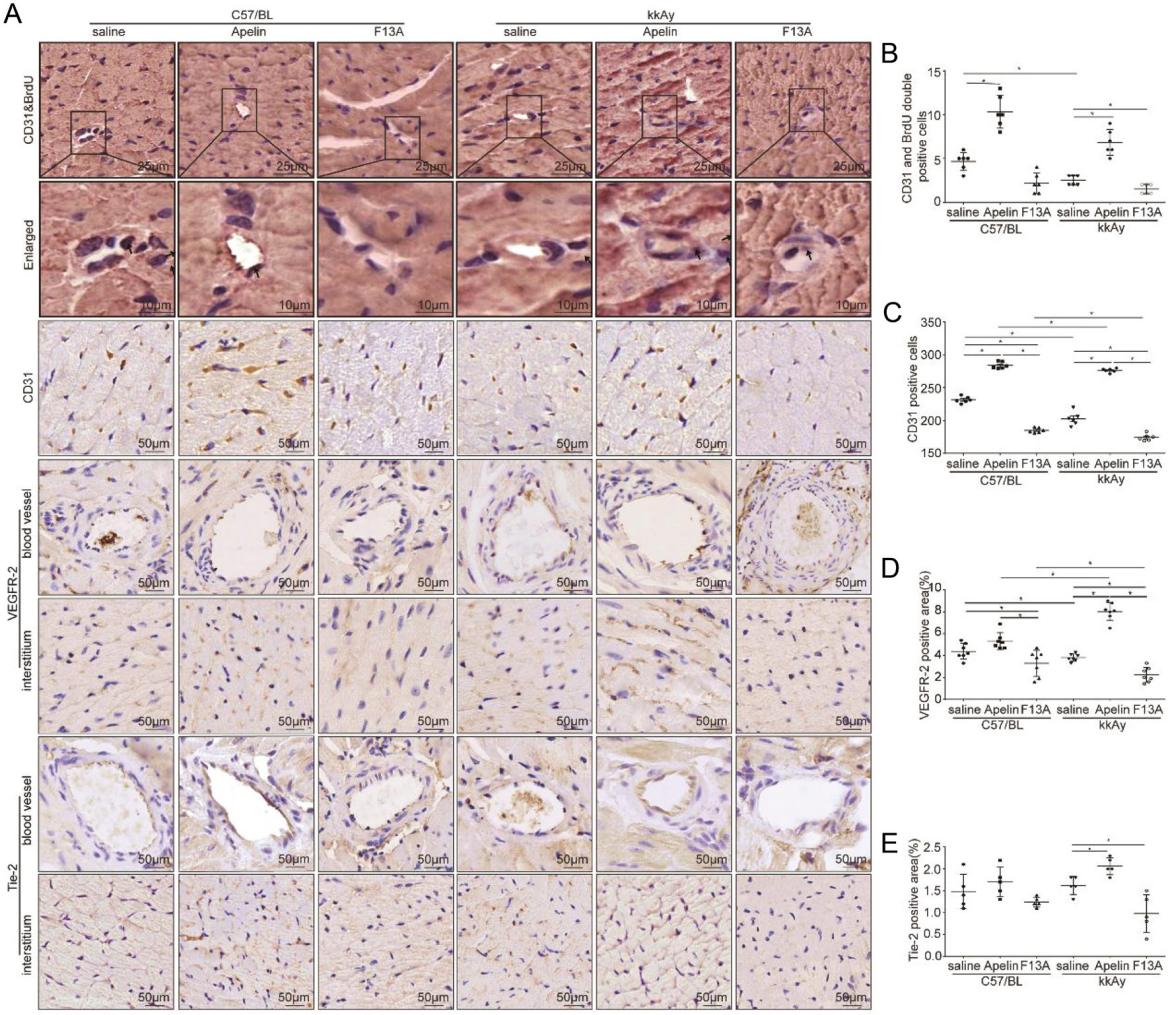
Apelin increased angiogenesis and proliferation of endothelial cells in heart of diabetic mice. (A) Representative images of immunohistochemistry for CD31&BrdU double staining, CD31,VEGFR-2 and Tie-2 in heart sections from C57 and diabetic mice with or without apelin/F13A treatment as quantified in (B, C, D and E) (CD31&BrdU: diabetes (*P* = 0.000), treatment (*P* = 0.000), interaction (*P* = 0.025); CD31: diabetes (*P* = 0.000), treatment (*P* = 0.000), interaction (*P* = 0.000); VEGFR-2: diabetes (*P* = 0.160), treatment (*P* = 0.000), interaction (*P* = 0.000); Tie-2: diabetes (*P* = 0.477), treatment (*P* = 0.000), interaction (*P* = 0.039); *n* = 6 mice per group, *P < 0.05). Scale bars represent 25, 10, nd 50 μm.

As the marker of endothelial cells, CD31 positive represents the changes in capillary density (Alasvand *et al.* 2016). Therefore CD31/BrdU double staining was used to investigate the effects of apelin on the proliferation of endothelial cells in the heart of diabetic mice. The results showed that the number of proliferated endothelial cells in kkAy mice (2.50 ± 0.54) was significantly reduced than that in C57 mice (4.67 ± 1.03) (diabetic effects, *P* < 0.01), which was significantly increased by apelin (10.33 ± 1.86 in apelin treated C57 mice and 6.83 ± 1.47 in apelin treated kkAy mice) both in C57 and kkAy mice (significant interaction between apelin and diabetic conditions, *P* < 0.01, *n* = 6; *P* < 0.05, Fig. 4A and  B). And the results from CD31 staining showed that CD31-positive cells in myocardial tissue of kkAy mice (202.50 ± 4.17) was significantly lower (diabetic effects, *P* < 0.01) than that of C57 mice (231.50 ± 1.99), which was significantly increased by apelin (284.00 ± 2.12 in apelin treated C57 mice and 276.33±1.28 in apelin treated kkAy mice) and further decreased by F13A (185.00 ± 1.52 in F13A treated C57 mice and 174.17 ± 2.08 in F13A treated kkAy mice) in kkAy mice (significant interaction between apelin/F13A and diabetic conditions, *P* < 0.01, *n* = 6; *P* < 0.05, Fig. 4A and  C). Meanwhile, the results showed that apelin significantly increased the expression of VEGFR2 (from 3.91 ± 0.14% to 7.93 ± 0.28%, Fig. 4A and  D) and tie-2 (from 1.75 ± 0.15% to 1.83 ± 0.17%) in heart of kkAy mice (significant interaction between apelin and diabetic conditions, *P* < 0.05; *n* = 6; *P* < 0.05, Fig. 4A and  E). These results indicated that apelin promoted the proliferation and angiogenesis of endothelial cells in heart of diabetic mice and improved microvascular density.

### Apelin decreased expression of adhesion molecules in arterioles of heart in diabetic mice

**Figure 5:**
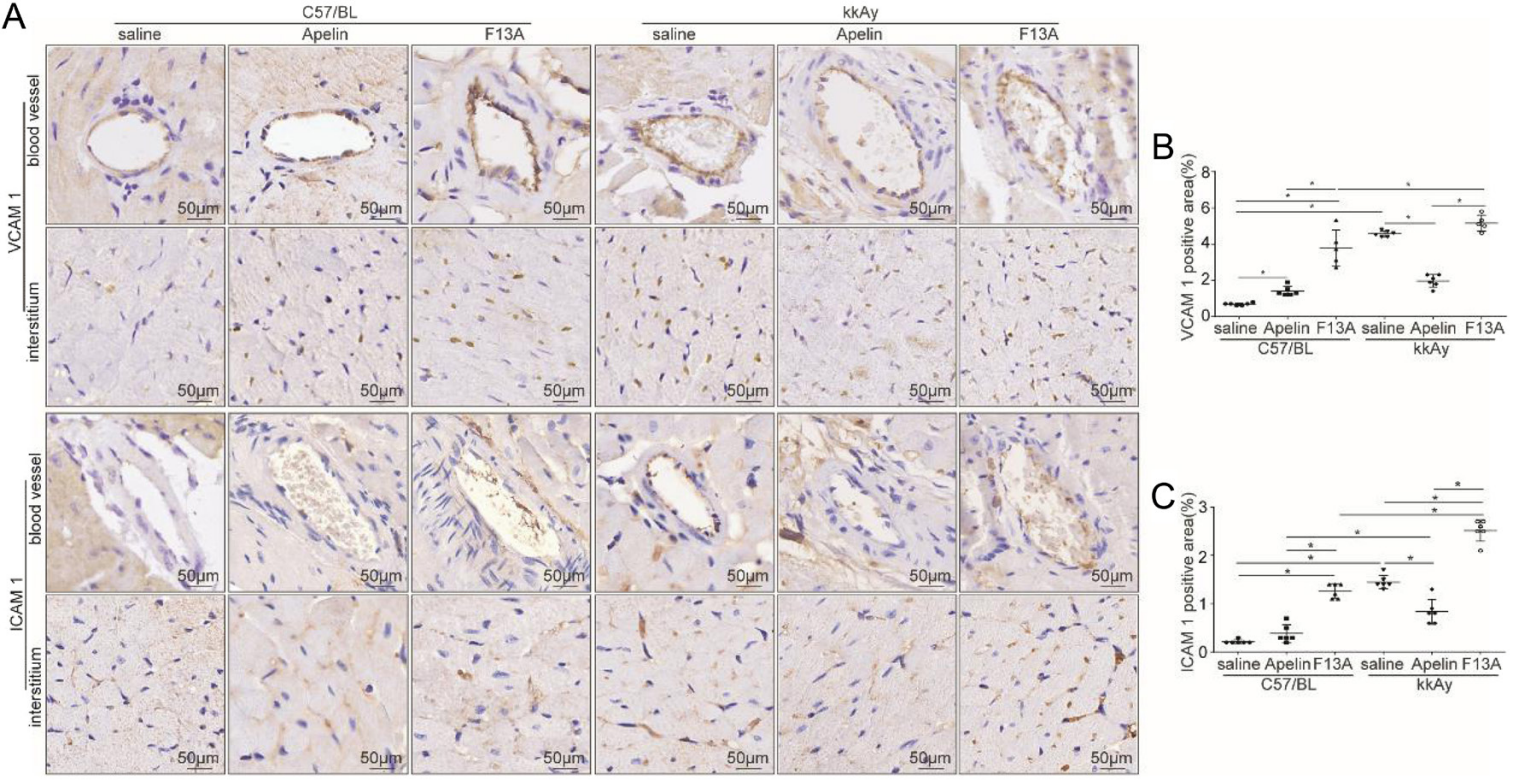
Apelin decreased adhesion molecules in heart of diabetic mice. (A) Representative images of immunohistochemistry for VCAM-1 and ICAM-1 in heart sections from C57 and diabetic mice with or without apelin/F13A treatment as quantified in (B and C) (VCAM-1: diabetes (*P* = 0.000), treatment (*P* = 0.000), interaction (*P* = 0.000); ICAM-1: diabetes (*P* = 0.000), treatment (*P =* 0.000), interaction (*P* = 0.000); *n*= 6 mice per group, *P < 0.05). Scale bars represent 50 μm.

Adhesive molecules have been reported to mediate the penetration of white blood cells from blood vessels. To verify the effects of apelin on permeability of arterioles in the heart from diabetic mice, immunostaining was used to detect the expression of ICAM-1 and VCAM-1. The results showed that the expression of ICAM-1 and VCAM-1 in hearts of kkAy mice (1.33 ± 0.04% and 4.26 ± 0.51%) was significantly increased (diabetic effects, *P* < 0.01) compared to that of C57 mice (0.23 ± 0.02% and 0.62 ± 0.04%), which was significantly reduced by apelin (1.09 ± 0.13% and 1.94 ± 0.13%) and further increased by F13A (2.92 ± 0.23% and 6.32 ± 0.63%, Fig. 5A, B and  C) in kkAy mice (significant interaction between apelin/F13A and diabetic conditions, *P* < 0.01, *n* = 6; *P* < 0.05). This result indicated that apelin may reduce the inflammatory response in hearts of diabetic mice via inhibiting expression of adhesion molecules in the endothelial cells of arterioles.

### Apelin improved endothelial dysfunction in high-glucose environment

**Figure 6:**
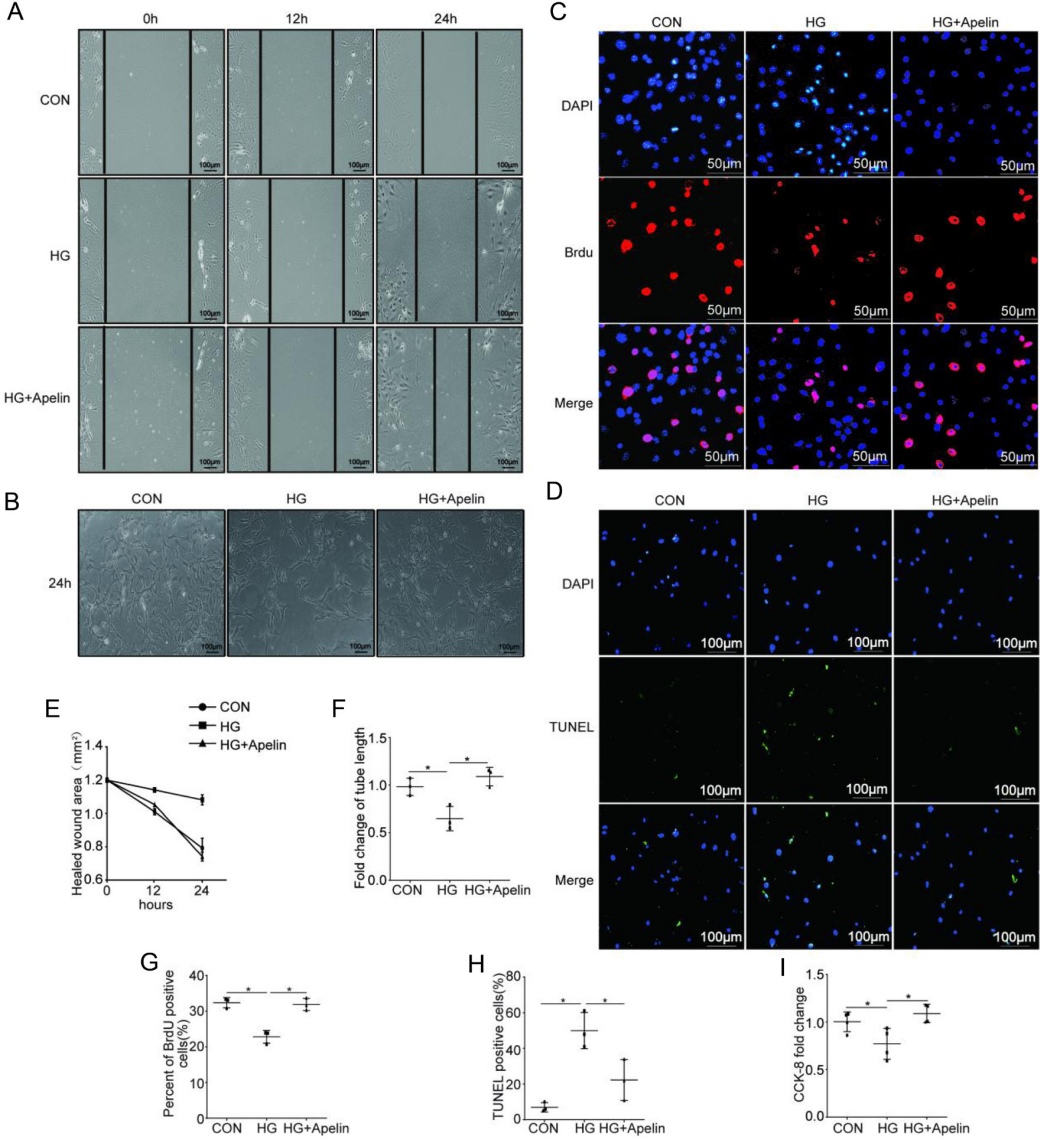
Apelin improved endothelial dysfunction in high-glucose environment. (A) Representative images of wound healing assay as quantified in (E). Scale bars represent 100 μm. (B) Representative images of Matrigel assay as quantified in (F) (*n* = 3, *P < 0.05). Scale bars represent 100 μm. (C) Representative images of the incorporation of BrdU as quantified in (G) (*n* = 3, *P < 0.05). Scale bars represent 50 μm. (D) Representative images of TUNEL assay as quantified in (H) (*n* = 3, *P < 0.05). Scale bars represent 100 μm. (I) Quantification of CCK-8 assay. *n* = 3, *P < 0.05.

To evaluate the effects of apelin on the endothelial migration in diabetic conditions, wound healing assay was used. The results showed that the healed wound areas of NCMECs was significantly larger in high glucose environment (1.08 ± 0.03 mm^2^) compared to that in control group (0.79 ± 0.05 mm^2^), which was decreased by apelin (0.74 ± 0.02 mm^2^) after 24-h treatment (*n* = 3; *P* < 0.05, Fig. 6A and  E).

To evaluate the effects of apelin on endothelial angiogenesis, tube formation of NCMECs in matrigel was analyzed. The results showed that the tube length formed by NCMECs under the high-glucose environment was significantly decreased to 0.69 folds than that in the control group, which was significantly increased to 1.07 folds by apelin (*n* = 3; *P* < 0.05, Fig. 6B and  F). These results indicated that apelin might increase angiogenesis via promoting the tube formatting ability of NCMECs in diabetic conditions.

To evaluate the effects of apelin on endothelial proliferation, BrdU incorporation and CCK-8 assay were used. The results showed that high glucose significantly inhibited the proliferation of NCMECs (from 35.71 ± 3.41% to 27.85 ± 2.05%), which was reversed by apelin (from 27.85 ± 2.05% to 31.13 ± 3.48%, *n* = 3; *P* < 0.05, Fig. 6C and  G). The results from CCK-8 assay showed similar results as BrdU (*n* = 3; *P* < 0.05, Fig. 6I). These results indicated that apelin might increase the angiogenesis in heart of diabetic mice via promoting proliferation of NCMECs.

To evaluate the effects of apelin on endothelial apoptosis in diabetic conditions, TUNEL staining was used. The showed that high glucose significantly increased the apoptosis of NCMECs (47.33 ± 3.01%), which was reversed by apelin (24.33 ± 3.37%, *n* = 3; *P* < 0.05, Fig. 6D and  H). These results indicated that apelin might increase the density of micro-vessels in hearts of diabetic mice via reducing apoptosis of NCMECs.

### Apelin relived endothelial dysfunction in diabetic conditions via NFκB pathway

**Figure 7:**
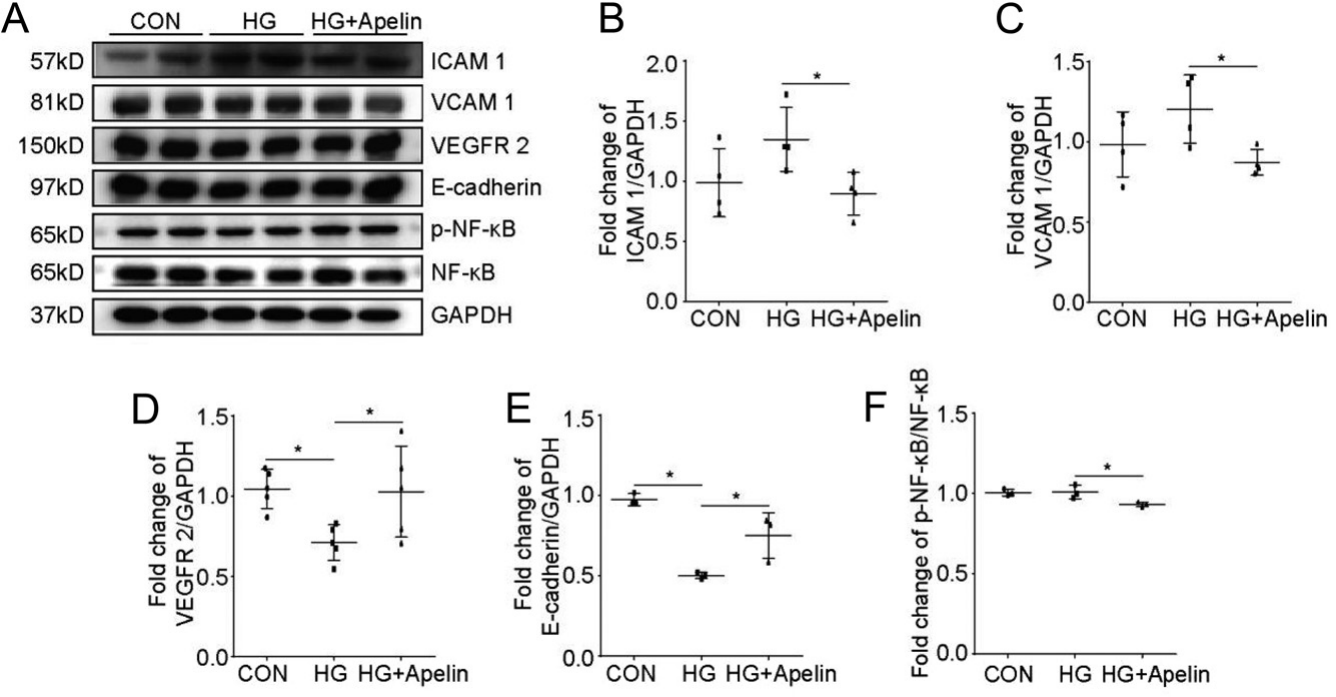
Apelin relived endothelial dysfunction dependent on NFκB pathway. (A) Representative images of western blot for ICAM-1, VCAM-1, VEGFR-2, E-cadherin, p-NFκB, NFκB and GAPDH after HG and apelin treatment in NCMECs. (B, C, D, E and F) Quantification of ICAM-1, VCAM-1, VEGFR-2, E-cadherin, and p-NFκB/NFκB in (A). Values represent the mean ± s.d. *n* = 3, *P < 0.05.

To investigate the intracellular pathways of apelin in reliving endothelial dysfunction, western blot was used to analyze the cell signaling pathways in NCMECs. The results showed that apelin significantly decreased the expression of ICAM-1 (from 1.06 folds to 0.69 folds, *n* = 3* P* < 0.05, Fig. 7A and  B) and VCAM-1 (from 1.20 folds to 0.84 folds, *n* = 3* P* < 0.05, Fig. 7A and  C) in NCMECs under high glucose environment compared to that in control groups. At the same time, apelin significantly promoted the expression of VEGFR2 (from 0.67 folds to 1.05 folds, *n* = 3* p* < 0.05, Fig. 7A and  D) and E-cadherin (from 0.51 folds to 0.83 folds, *n* = 3* P* < 0.05, Fig. 7A and  E) in NCMECs under high glucose environment compared to that in control groups. These results confirmed that apelin might improve endothelial dysfunction in diabetic condition via decreasing expression of adhesive molecules and increasing expression of VEGFR2 and E-cadherin in endothelial cells.

To verify the cell signaling pathways mediated the alleviating effects of apelin on endothelial dysfunction in diabetic conditions, NFκB pathway was detected with western blot. The results showed that apelin decreased the phosphorylation of NFκB in high-glucose-treated NCMECs (from 1.01 folds to 0.92 folds, Fig. 7A and  F). These results suggested that apelin might be involved in protecting endothelial cells from injuries in diabetic condition by inhibiting the NFκB pathway.

### Effects of apelin on diabetic cardiomyopathy were dependent on APJ

To verify whether the effects of apelin on endothelial cells in diabetic condition were dependent on APJ, APJ was knockout in endothelial cells. The results from immunostaining showed that expression of APJ in endothelial cells of APJ^ΔEC^ mice were significantly decreased compared to that of APJ^fl/fl^ mice without significant influence on cardiomyocytes, smooth muscle cells or hepatocytes (Supplementary Fig. 3).

First of all, blood glucose was detected to confirm the successfulness of diabetic models. Compared to control mice (9.76 ± 1.91 mmol/L in APJ^fl/fl^mice, 9.46 ± 0.08 mmol/L in APJ^ΔEC^ mice, *n* = 6; *P*  < 0.05), the blood glucose in STZ treated mice were significantly increased (16.85 ± 0.72 mmol/L in APJ^fl/fl^ mice, 24.95 ± 1.61 mmol/L in APJ^ΔEC^ mice, *n* = 6; *P*  < 0.05), and apelin infusion decreased the blood glucose to 10.51 ± 0.25 mmol/L in STZ treated APJ^fl/fl^ mice and 19.96 ± 0.33 mmol/L in STZ treated APJ^ΔEC^ mice (*n* = 6; *P*  < 0.05, Supplementary Fig. 4).

**Figure 8:**
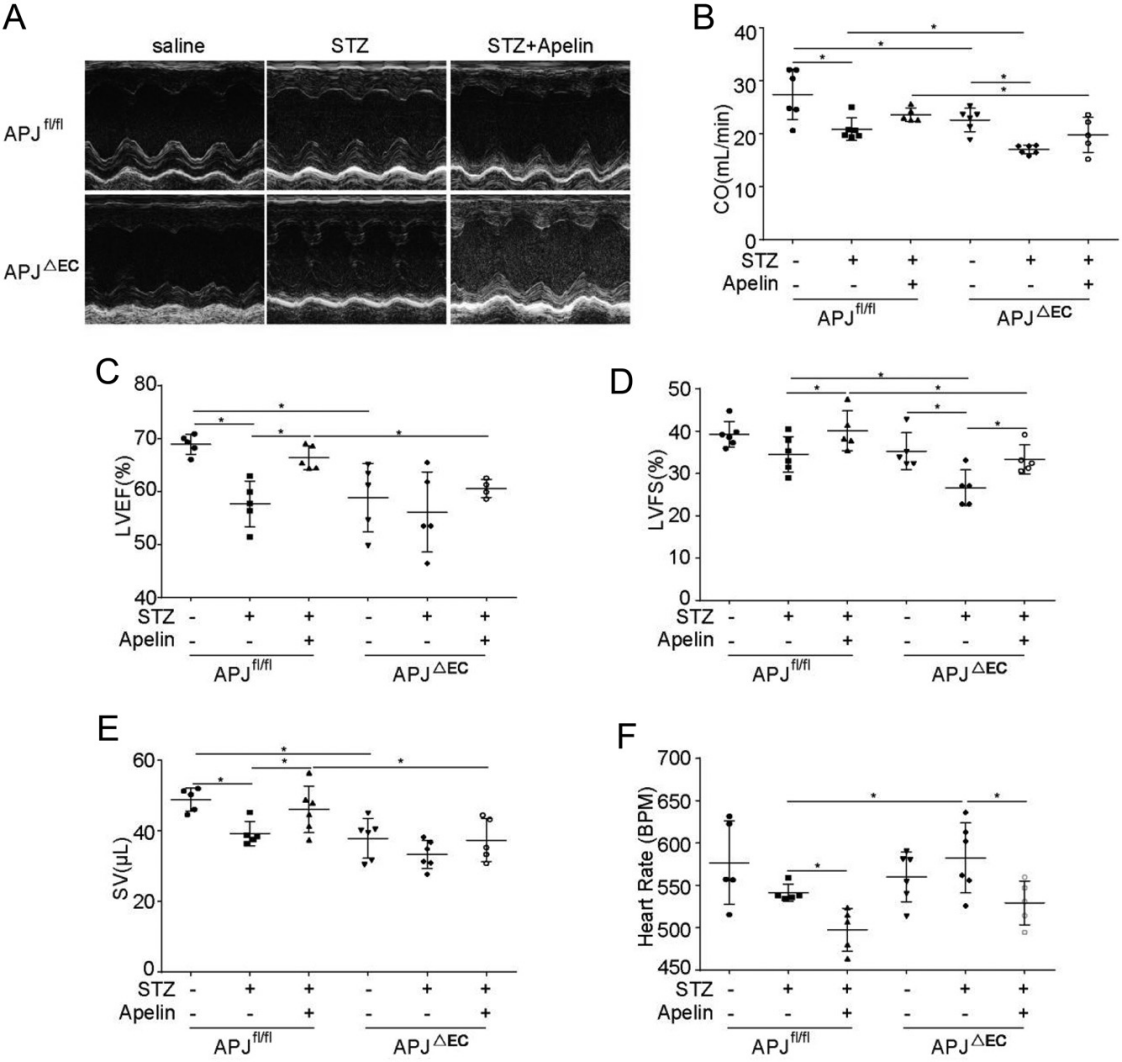
Effects of apelin on cardiac function of APJ^ΔEC^ mice. (A) The M-mode of echocardiography for each group. (B) Quantification of the cardiac output (CO). (C) Quantification of the left ventricular ejection fraction (LVEF). (D) Quantification of the left ventricular fractional shortening (LVFS). (E) Quantification of the stroke volume (SV). (F) Quantification of heart rate. *n* = 6 mice per group, *P < 0.05.

Secondly, the cardiac dysfunction induced by type 1 diabetes was detected. The results showed that the cardiac function of the APJ^ΔEC^ mice were significantly decreased compared to that of APJ^fl/fl^ mice (CO: 22.65 ± 0.91 mL/min in APJ^ΔEC^ mice vs 27.46 ± 1.94 mL/min in APJ^fl/fl^mice, LVEF: 63.28 ± 0.82% in APJ^ΔEC^ mice vs 70.28 ± 1.53% in APJ^fl/fl^mice, SV: 40.10 ± 0.28 μL in APJ^ΔEC^ mice vs 48.89 ± 1.47 μL in APJ^fl/fl^mice, *n*  = 6; *P*  < 0.05; Fig. 8). Meanwhile apelin significantly improved the LVEF (left ventrical ejection fration) and SV (stroke volume) in diabetic APJ^fl/fl^ mice (LVEF: from 57.67 ± 1.92% to 66.37 ± 1.00% and SV: from 39.35 ± 1.54 to 46.12 ± 2.69 μL, *n* = 6, *P* < 0.05; Fig. 8) but no such effects in APJ^ΔEC^ mice (LVEF: from 56.13 ± 3.37% to 60.57 ± 0.84% and SV: from 33.41 ± 1.65 to 37.44 ± 2.72 μL, *n* = 6, *P* > 0.05; Fig. 8). And apelin showed no significant effects on CO (cardiac output) on either diabetic APJ^fl/fl^ mice (from 20.89 ± 0.86 to 23.62 ± 0.57 mL/min, *n* = 6; *P* > 0.05, Fig. 8) or diabetic APJ^ΔEC^ mice (from 17.03 ± 0.34 to 19.82 ± 1.47 mL/min, *n* = 6; *P* > 0.05, Fig. 8), which might be related to the decreased heart rate by apelin both in diabetic APJ^fl/fl^ mice (from 541.27 ± 4.48 to 497.87 ± 11.27 BPM, *n* = 6; *P* < 0.05, Fig. 8) and diabetic APJ^ΔEC^ mice (from 582.49 ± 16.85 to 529.38 ± 11.55 BPM, *n* = 6; *P*  < 0.05, Fig. 8). And apelin significantly improved LVFS (left ventrical fractional shortening) both in diabetic APJ^fl/fl^ mice (from 34.51 ± 1.71% to 40.08 ± 2.10%, *n* = 6; *P* < 0.05, Fig. 8) and diabetic APJ^ΔEC^ mice (from 26.58 ± 1.90% to 33.32 ± 1.52%, *n* = 6; *P*  < 0.05, Fig. 8). Even though, APJ knockout in endothelial cells did decrease the improving effects of apelin on cardiac function in diabetic mice (CO: 19.82 ± 1.47 mL/min in apelin treated diabetic APJ^ΔEC^ mice vs 22.65 ± 0.91 mL/min in apelin treated diabetic APJ^fl/fl^ mice, LVEF: 60.57 ± 0.84% in apelin treated diabetic APJ^ΔEC^ mice vs 66.37 ± 1.01% in apelin treated diabetic APJ^fl/fl^ mice, LVFS: 33.32 ± 1.52% in apelin treated diabetic APJ^ΔEC^ mice vs 40.08 ± 2.10% in apelin treated diabetic APJ^fl/fl^ mice, SV: 37.44 ± 2.72 μL in apelin treated diabetic APJ^ΔEC^ mice vs 45.12 ± 2.69 μL in apelin treated diabetic APJ^fl/fl^ mice, *n* = 6; *P* < 0.05, Fig. 8).

**Figure 9:**
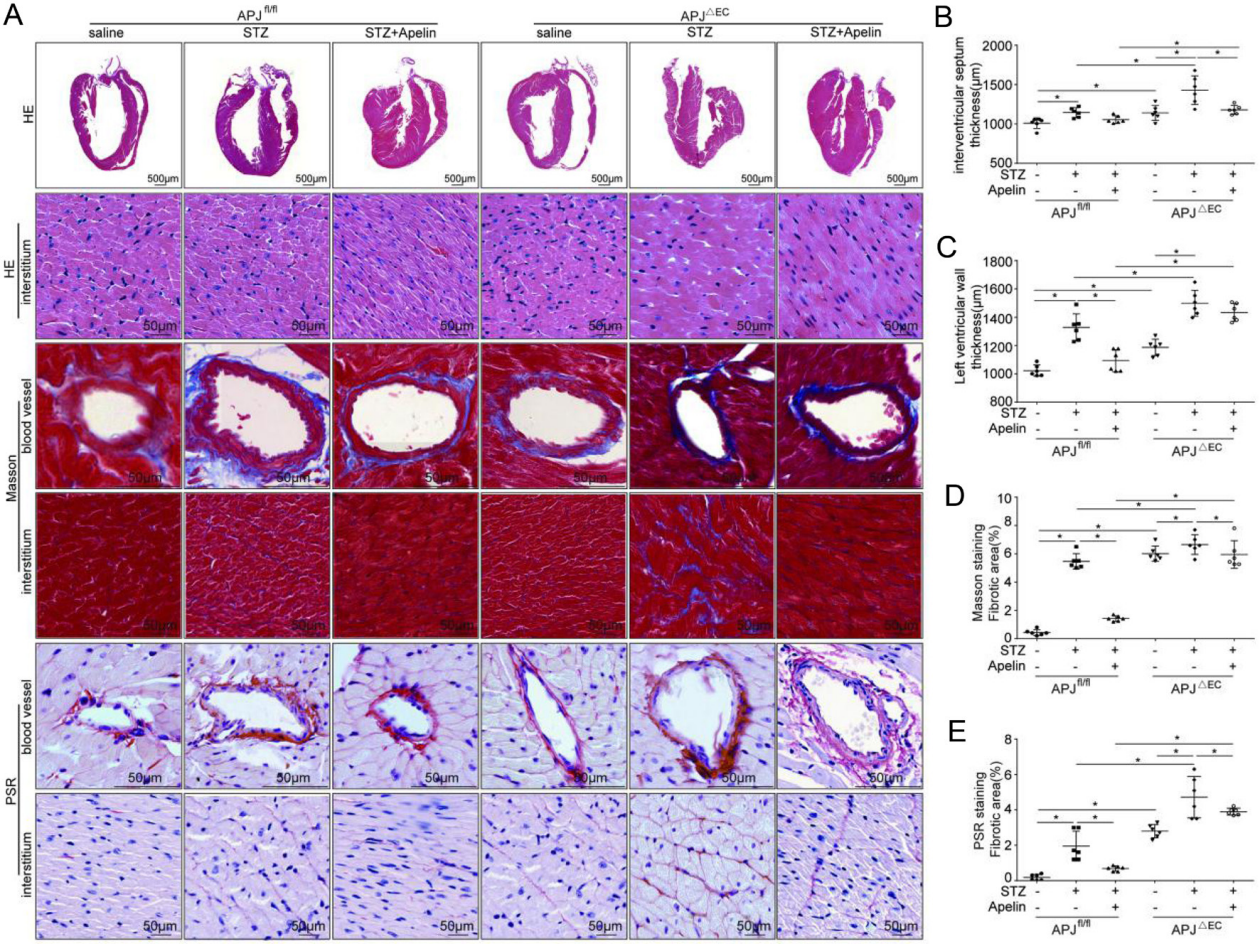
Effects of apelin on heart structure in diabetic APJ^ΔEC^ mice. (A) Representative HE histology, Masson Trichrome and Pico Sirius Red (PSR) staining in heart sections from APJ^fl/fl^and APJ^ΔEC^ mice with or without apelin treatment as quantified in (B, C, D and E) (*n* = 6 mice per group, *P < 0.05). Scale bars represent 50 μm.

The results from HE staining showed that the ventricular wall in APJ^ΔEC^ mice was significantly thicker than that in APJ^fl/fl^mice (septum thickness: 1140.25 ± 38.69 μm in APJ^ΔEC^ mice vs 1010.42 ± 26.84 μm in APJ^fl/fl^mice; left ventricular wall thickness: 1186.72 ± 11.26 μm in APJ^ΔEC^ mice vs 1022.91 ± 16.17 μm in APJ^fl/fl^mice, *n* = 6; *P* < 0.05), which was increased in diabetic conditions(septum thickness: 1144.91 ± 25.28 μm in diabetic APJ^fl/fl^ mice vs 1010.42 ± 26.84 μm in APJ^fl/fl^ mice, 1430.14 ± 74.55 in diabetic APJ^ΔEC^ mice vs 1140.25 ± 38.69 μm in APJ^ΔEC^ mice, left ventricular wall thickness: 1327.62 ± 39.12 in diabetic APJ^fl/fl^ mice vs 1022.91 ± 16.17 μm in APJ^fl/fl^ mice, 1499.15 ± 14.03 μm in diabetic APJ^ΔEC^ mice vs.1186.72 ± 11.26 μm in APJ^ΔEC^ mice, *n* = 6; *P* < 0.05). And APJ knockout in endothelial cells significantly decreased the inhibiting effects of apelin on the ventricular wall in diabetic mice (septum thickness: 1179.26 ± 23.09 μm in apelin treated diabetic APJ^ΔEC^ mice vs 1054.72 ± 19.90 μm in apelin treated diabetic APJ^fl/fl^ mice; left ventricular wall thickness: 1434.12 ± 25.52 μm in apelin treated diabetic APJ^ΔEC^ mice vs 1093.81 ± 10.13 μm in apelin treated diabetic APJ^fl/fl^ mice, *n* = 6; *P* < 0.05, Fig. 9A, B and  C).

The results from masson and PSR staining showed that the myocardial fibrosis in APJ^ΔEC^ mice was significantly higher than that in APJ^fl/fl^ mice (Masson: 6.01 ± 0.22% in APJ^ΔEC^ mice vs 0.43 ± 0 .08% in APJ^fl/fl^mice, PSR: 2.80 ± 0.15% in APJ^ΔEC^ mice vs 0.18 ± 0.06% in APJ^fl/fl^mice, *n* = 6; *P* < 0.05), which was increased in diabetic conditions (Masson: 5.46 ± 0.22% in diabetic APJ^fl/fl^ mice vs 0.43 ± 0.08% in APJ^fl/fl^ mice, 6.65 ± 0.28% in diabetic APJ^ΔEC^ mice vs 6.01 ± 0.22% in APJ^ΔEC^ mice, PSR: 1.95 ± 0.34% in diabetic APJ^fl/fl^ mice vs 0.18 ± 0.06% in APJ^fl/fl^ mice, 4.71 ± 0.47% in diabetic APJ^ΔEC^ mice vs 2.80 ± 0.15% in APJ^ΔEC^ mice, *n* = 6; *P*  < 0.05). At the same time, APJ knockout in endothelial cells significantly decreased the inhibiting effects of apelin on fibrosis in the myocardial tissue of diabetic mice (Masson: 5.94 ± 0.39% in apelin treated diabetic APJ^ΔEC^ mice vs 1.42 ± 0.07% in apelin treated diabetic APJ^fl/fl^mice, PSR: 3.89 ± 0.08% in apelin treated diabetic APJ^ΔEC^ mice vs 0.68 ± 0.06% in apelin treated diabetic APJ^fl/fl^mice, *n* = 6; *P*  < 0.05, Fig. 9).

**Figure 10:**
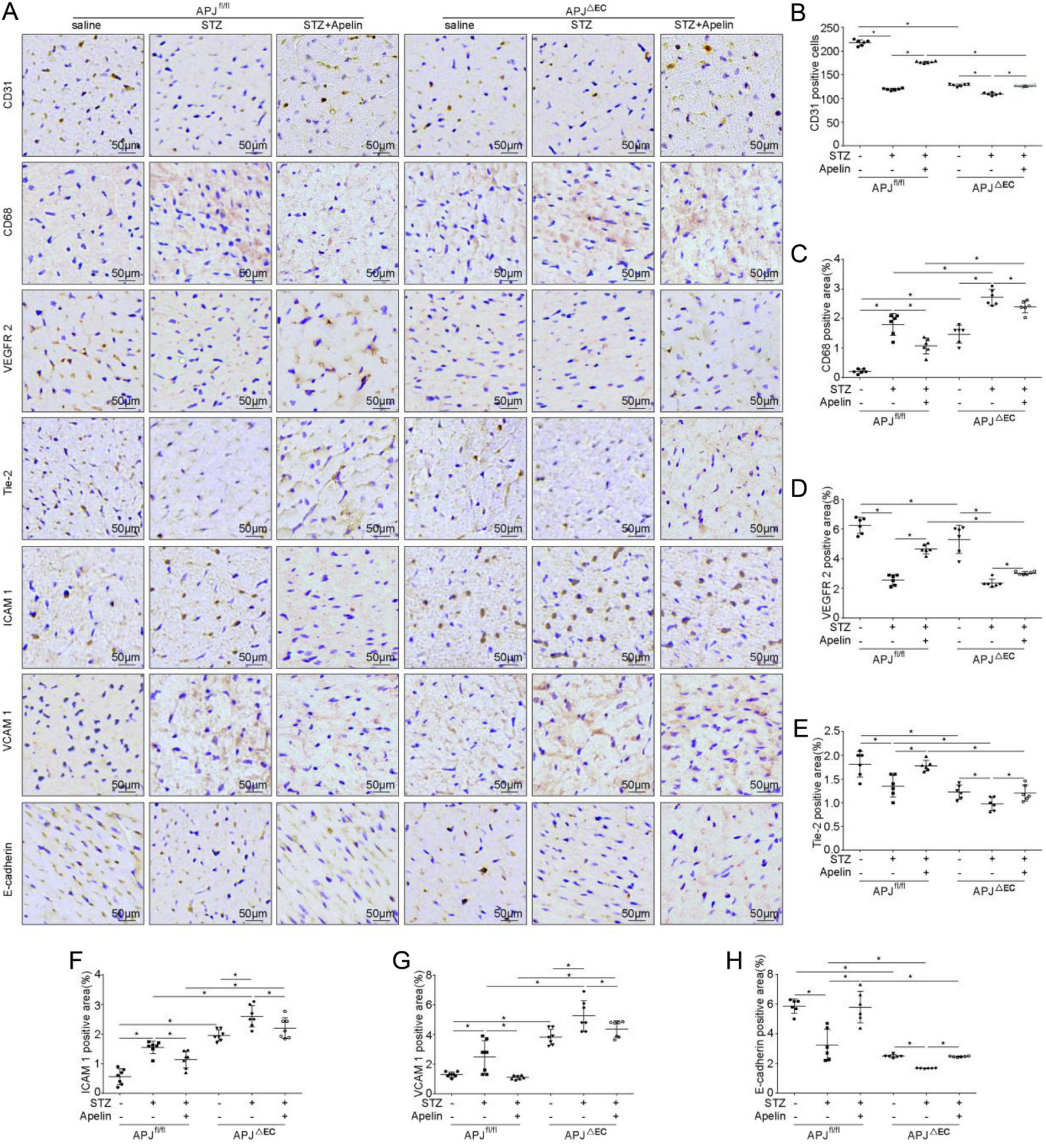
Effects of apelin on endothelial dysfunction in diabetic APJ^ΔEC^ mice. (A) Representative images of immunohistochemistry for CD31, CD68, VEGFR-2, Tie-2, ICAM-1, VCAM-1 and E-cadherin in heart sections from APJ^fl/fl^ and APJ^ΔEC^ mice with or without apelin treatment as quantified in (B, C, D, E, F, G and H) (*n* = 6 mice per group, *P < 0.05). Scale bars represent 50 μm.

Results from CD31 staining showed that CD31-positive cells in the myocardial tissue of APJ^ΔEC^ mice were significantly lower than that of APJ^fl/fl^ mice (127.67 ± 1.11 in APJ^ΔEC^ mice vs 217.50 ± 2.43 in APJ^fl/fl^mice, *n* = 6; *P* < 0.05), which was reduced in diabetic condition (119.33 ± 0.88 in diabetic APJ^fl/fl^ mice vs 217.50 ± 2.43 in APJ^fl/fl^ mice, 109.50 ± 1.17 in diabetic APJ^ΔEC^ mice vs 127.67 ± 1.11 in APJ^ΔEC^ mice, *n* = 6; *P* < 0.05). Meanwhile, APJ knockout in endothelial cells significantly decreased the increasing effects of apelin on the number of CD31-positive cells in myocardial tissue in diabetic mice (176.50 ± 0.76 in apelin treated diabetic APJ^fl/fl^mice vs 126.17 ± 0.83 in apelin treated diabetic APJ^ΔEC^ mice, *n* = 6; *P* < 0.05, Fig. 10A and B).

The results from CD68 staining showed that CD68-positive cells in myocardial tissue of APJ^ΔEC^ mice were significantly more than that in APJ^fl/fl^ mice (1.46 ± 0.12% in APJ^ΔEC^ mice vs 0.21 ± 0.03% in APJ^fl/fl^mice, *n* = 6; *P*  < 0.05), which were increased in diabetic conditions (1.80 ± 0.15% in diabetic APJ^fl/fl^ mice vs 0.21 ± 0.03% in APJ^fl/fl^ mice, 2.71 ± 0.10% in diabetic APJ^ΔEC^ mice vs 1.46 ± 0.12% in APJ^ΔEC^ mice, *n* = 6; *P* < 0.05). On the other hand, APJ knockout in endothelial cells significantly decreased the reducing effects of apelin on the CD68 positive cells in cardiac tissue of diabetic mice (1.07 ± 0.11% in apelin treated diabetic APJ^fl/fl^mice vs 1.80 ± 0.15% in apelin treated diabetic APJ^ΔEC^ mice, *n* = 6; *P*  < 0.05, Fig. 10A and C).

Results from Tie-2 and VEGFR-2 staining showed that the expression of Tie-2 and VEGFR-2 in the myocardial tissue of APJ^ΔEC^ mice was significantly lower than that in APJ^fl/fl^ mice (VEGFR-2: 5.31 ± 0.38% in APJ^ΔEC^ mice vs 6.25 ± 0.22% in APJ^fl/fl^mice, Tie-2: 1.22 ± 0.06% in APJ^ΔEC^ mice vs 1.81 ± 0.11% in APJ^fl/fl^mice, *n* = 6; *P* < 0.05), which was reduced in diabetic condition (VEGFR-2: 2.57 ± 0.14% in diabetic APJ^fl/fl^ mice vs 6.25 ± 0.22% in APJ^fl/fl^ mice, 2.35 ± 0.11% in diabetic APJ^ΔEC^ mice vs 5.31 ± 0.38% in APJ^ΔEC^ mice, Tie-2: 1.35 ± 0.09% in diabetic APJ^fl/fl^ mice vs 1.81 ± 0.11% in APJ^fl/fl^ mice, 0.97 ± 0.05% in diabetic APJ^ΔEC^ mice vs 1.22 ± 0.06% in APJ^ΔEC^ mice, *n* = 6; *P* < 0.05). Meanwhile, APJ knockout in endothelial cells significantly decreased the increasing effects of apelin on the expression of Tie-2 and VEGFR-2 in myocardial tissue of diabetic mice (VEGFR-2: 3.03 ± 0.04% in apelin treated diabetic APJ^fl/fl^mice vs 4.65 ± 0.12% in apelin treated diabetic APJ^ΔEC^ mice, Tie-2: 1.20 ± 0.06% in apelin treated diabetic APJ^fl/fl^mice vs 1.78 ± 0.04% in apelin treated diabetic APJ^ΔEC^ mice, *n* = 6; *P* < 0.05, Fig. 10A, D and E).

The results from immunostaining showed that expression of ICAM-1 and VCAM-1 in myocardial tissue of APJ^ΔEC^ mice were significantly higher than that of APJ^fl/fl^ mice (ICAM- 1: 1.95 ± 0.07% in APJ^ΔEC^ mice vs 0.55 ± 0.09% in APJ^fl/fl^mice, VCAM 1: 3.81 ± 0.20% in APJ^ΔEC^ mice vs 1.78 ± 0.06% in APJ^fl/fl^mice, *n* = 6; *P* < 0.05), which were increased in diabetic condition (ICAM-1: 1.54 ± 0.08% in diabetic APJ^fl/fl^ mice vs 0.55 ± 0.09% in APJ^fl/fl^ mice, 2.60 ± 0.14% in diabetic APJ^ΔEC^ mice vs 1.95 ± 0.07% in APJ^ΔEC^ mice, VCAM-1: 2.48 ± 0.41% in diabetic APJ^fl/fl^ mice vs 1.78 ± 0.06% in APJ^fl/fl^ mice, 5.25 ± 0.38% in diabetic APJ^ΔEC^ mice vs 3.81 ± 0.20% in APJ^ΔEC^ mice, *n* = **6; *P* < 0.05). Meanwhile, APJ knockout in endothelial cells significantly decreased the reducing effects of apelin o n the expression of ICAM-1 (2.19 ± 0.13% in apelin treated diabetic APJ^ΔEC^ mice vs 1.14 ± 0.10% in apelin treated diabetic APJ^fl/fl^mice, *n* = 6; *P*  < 0.05) and VCAM-1 (4.36 ± 0.20% in apelin treated diabetic APJ^ΔEC^ mice vs 1.09 ± 0.04% in apelin treated diabetic APJ^fl/fl^mice, *n* = 6; *P* < 0.05) in diabetic mice. At the same time, the expression of E-cadherin in the myocardial tissue of APJ^ΔEC^ mice was significantly lower than that of APJ^fl/fl^ mice (2.49 ± 0.04% in APJ^ΔEC^ mice vs 5.86 ± 0.19% in APJ^fl/fl^mice, *n* = 6; *P* < 0.05), which was decreased in diabetic conditions (3.23 ± 0.43% in diabetic APJ^fl/fl^ mice vs 5.86 ± 0.19% in APJ^fl/fl^ mice, 1.68 ± 0.01% in diabetic APJ^ΔEC^ mice vs 2.49 ± 0.04% in APJ^ΔEC^ mice, *n* = 6; *P* < 0.05). APJ knockout in endothelial cells significantly decreased the increasing effects of apelin on the expression of E-cadherin in diabetic mice (2.46 ± 0.02% in diabetic APJ^ΔEC^ mice with apelin vs 5.80 ± 0.43% in diabetic APJ^fl/fl^mice with apelin, *n* = 6; *P* < 0.05, Fig. 10A, F, G and H).

## Discussion

In this study, it is revealed that apelin relived diabetic cardiomyopathy (Figs 1 and  2) via increasing angiogenesis (Fig. 4) and decreasing endothelial dysfunction (Figs 3, 5 and  6), which were dependent on endothelial APJ activated NFκB pathway(Figs 7, 8, 9 and  10). Meanwhile, these effects might not exist in normal hearts.

First of all, the effects of apelin on cardiac function of diabetic mice were analyzed. The results showed that apelin infusion ameliorated cardiac dysfunction in diabetic mice (Fig. 1), which means that apelin protected the heart from cardiac dysfunction induced by diabetes mellitus. These results were supported by previous results which reported that apelin protected heart from injuries induced by ischemia, reperfusion (Zhang *et al.* 2014) or hypertension (Sekerci *et al.* 2018). If the mechanisms are similar to previous reports, would there be similar protective effects on heart injuries induced by diabetes mellitus?

Therefore, the morphological changes in hearts of diabetic mice were detected. The results showed that apelin decreased cardiac hypertrophy, glycogen deposition and fibrosis in heart of diabetic mice (Fig. 2), which confirmed that apelin protected the heart from diabetic injuries. As being reported, fibrosis might be caused by inflammation in cardiac tissue induced by high glucose (Mack 2018). Would apelin reduce the inflammatory response in hearts in diabetic conditions?

Thirdly, the inflammatory responses in hearts were analyzed. The results showed that CD68, a lysosomal marker for activated macrophages (Ni *et al.* 2019), was significantly decreased by apelin in hearts of diabetic mice (Fig. 3A and  B). These results indicated that apelin involved in the inflammatory response of heart during diabetes via decreasing the infiltration of macrophages. Interestingly, E-cadherin, the tight junction between endothelial cells, was significantly increased by apelin in heart of diabetic mice (Fig. 3A and  D), which indicated that apelin might inhibit the macrophage infiltration in heart of diabetic mice via increasing tight junction between endothelial cells. These were consistent with reports that endothelial E-cadherin regulate vascular permeability (Yang *et al.* 2008, Sidibe & Imhof 2014).

To confirm the effects of apelin on phagocytosis, mac-2, a galactose-specific lectin whose expression indicated the processing phagocytosis (Rotshenker 2009) and inversely correlated to fibrosis in patients with inflammatory cardiomyopathy (Besler *et al.* 2017), was detected. The results showed that apelin significantly increased expression of mac-2 in hearts of diabetic mice (Fig. 3A and  C). These results indicated that apelin not only affected the endothelial function but also the function of macrophages in heart of diabetic mice. As E-cadherin was reported to be also involved in angiogenesis (Pinheiro *et al.* 2012, Tang *et al.* 2018) and endothelial proliferation (Vestweber 2008, Mao *et al.* 2017), is apelin involved in these process too in diabetic conditions?

Consequently, angiogenesis and proliferation of endothelial cells were analyzed in heart of diabetic mice after apelin treatment. The results showed that proliferated endothelial cells, which were both CD31 and BrdU positive, were increased in heart of diabetic mice with apelin treatment. Meanwhile, the result from CD31 staining showed that apelin significantly increased capillary density in heart of diabetic mice. In addition, apelin showed the same effects on expression of VEGFR-2 and Tie-2 in hearts of diabetic mice (Fig. 4A, D and  E). These results indicated that apelin might promote the angiogenesis via promoting proliferation of endothelial cells dependent on VEGFR-2 and Tie-2 pathway, which were consistent with previous reports (Zhang *et al.* 2013). Then, how did the angiogenesis and proliferation of endothelial cells affect inflammatory response?

As adhesion molecules have been reported to be related to the permeability of microvascular (Zhang *et al.* 2013, Reglero-Real *et al.* 2016), ICAM-1 and VCAM-1 were detected in heart of diabetic mice with or without apelin treatment. The results showed that the expression of ICAM-1 and VCAM-1 was significantly increased in heart of diabetic mice, which was reversed by apelin (Fig. 5A, B and  C). These results indicated that apelin might reduce the permeability of microvascular endothelial cells by decreasing the expression of ICAM-1 and VCAM-1 in cardiac microvascular endothelial cells. Then, is apelin affected the endothelial cells directly or indirectly to regulating the microvascular functions in hearts of diabetic mice?

To confirm the effects of apelin on endothelial cells in diabetic mice, native cardiac endothelial cells (NCMECs) were cultured, and high glucose were applied to mimic the diabetic condition. The results showed that apelin significantly increased expression of VEGFR-2 and E-cadherin under high glucose conditions (Fig. 7A, D and  E), and the proliferation, migration and tube formation of NCMECs were increased as well under high-glucose treatment (Fig. 6A, B, C, E, F, G and  I). At the same time, apelin significantly reduced apoptosis (Fig. 6D and  H) and the expression of ICAM-1 and VCAM-1 of NCMECs in high glucose environment (Fig. 7A, B and  C). These results indicated that apelin attenuates the microvascular dysfunction induced by hyperglycemia via promoting endothelial proliferation and decreasing apoptosis and expression of adhesion molecules in endothelial cells.

As NFκB has been reported to mediate the transcription of adhesion molecules (Tang *et al.* 2016, Zhong *et al.* 2018), the effects of apelin on NFκB were analyzed in endothelial cells to investigate the intracellular pathways induced by apelin. The results showed that phosphorylation of NFκB was increased in high-glucose-treated NCMECs, which were reversed by apelin (Fig. 7A and  F). These results suggested that apelin might inhibited the expression of ICAM-1 and VCAM-1 in endothelial cells under high glucose condition via inactivating NFκB pathway.

Then the question remained was, were all these effects of apelin dependent on the endothelial receptor APJ? Therefore endothelial specific APJ knockout mice were adopted to detect the effects of apelin on diabetic cardiomyopathy. The results showed that all the effects of apelin on endothelial cell dysfunction, cardiac dysfunction and cardiac morphological changes in diabetic mice were partially canceled by specific knockout of APJ in endothelial cells (Figs 8, 9 and  10). These results supported the idea that APJ mediated the protective effect of apelin on relieving diabetic cardiomyopathy by reducing microvascular dysfunction.

However, it must be noted that apelin infusion decreased the blood glucose in diabetic mice which was partially canceled by APJ knockout in endothelial cells as shown in S2 and S3. These results means that apelin may protect the heart from diabetic cardiomyopathy partially dependent on decreasing blood glucose. And APJ knockout in endothelial cells did not totally inhibited the effects of apelin on diabetic cardiomyopathy including microvascular or endothelial dysfunctions, which means that apelin may showed effects in an APJ independent way or other cells such as cardiomyocyte expressing APJ involved the attenuating effects of apelin too.

## Conclusions

Apelin alleviated diabetic cardiomyopathy via attenuating endothelial cell dysfunction induced by diabetes mellitus, including increasing proliferation, migration, tube formation and decreasing apoptosis, expression of adhesion molecules. Meanwhile, the effects of apelin on endothelial cells were partially dependent on APJ activated NFκB pathway. These results might provide a potential pathway for treatment of diabetic cardiomyopathy.

## Supplementary Material

Figure S1. Expression of APJ in myocardial tissue and blood vessels. Representative images of immunohistochemistry for APJ in heart sections from C57 and diabetic mice as quantified in (B) (n=6 mice per group, *p< 0.05). Scale bars represent 50μm.

Figure S2. Random blood glucose after 4 weeks’ treatment in C57 and diabetic mice. Representative random blood glucose from C57 and diabetic mice with or without apelin/F13A treatment as quantified using unpaired Student’s t-test (n=6 mice per group, *p< 0.05).

Figure S3. Expression of APJ in APJfl/fl and APJ△EC mice. Representative images of immunohistochemistry for APJ in heart, liver, glomeruli and artery sections from APJfl/fl and APJ△EC mice. Scale bars represent 50μm.

Figure S4. Random blood glucose after 4 weeks’ treatment in APJfl/fl and APJ△EC mice. Representative random blood glucose from APJfl/fl and APJ△EC mice with or without apelin treatment as quantified using unpaired Student’s t-test (n = 6 mice per group, *p< 0.05).

## Declaration of interest

The authors declare that there is no conflict of interest that could be perceived as prejudicing the impartiality of the research reported.

## Funding

This work was supported by the Natural Science Foundation of China Grant No. 81270815, No. 81500320 and No. 32071112. This work was also partially supported by Beijing Municipal Commission of Education General project. No. KM201910025028.

## Author contribution statement

Xiangjun Zeng designed the study; Bin Li analyzed the data; Bin Li, Jiming Yin, Jing Chang, Jia Zhang and Yangjia Wang performed the experiments; Haixia Huang, Wei Wang and X. Zeng gave suggestions for the study and critically revised the manuscript; Bin Li and Xiangjun Zeng wrote the manuscript; and Xiangjun Zeng got the funding.
