# Supplementary figures and images for: Apelin/APJ relieve diabetic cardiomyopathy by reducing microvascular dysfunction
Source: J Endocrinol. 2021 Jan 25;249(1):1–18. doi: 10.1530/JOE-20-0398 (PMC8052525; doi:10.1530/JOE-20-0398)

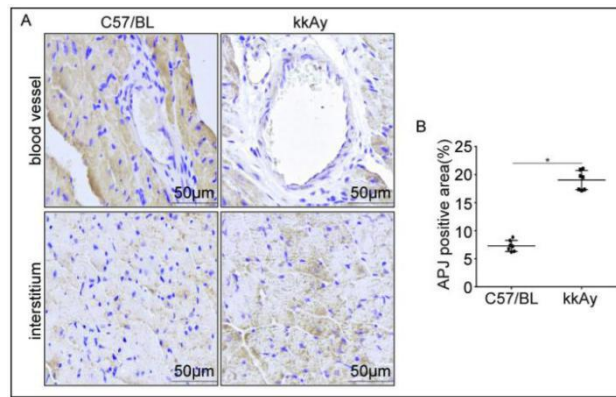

**Figure S1.** Expression of APJ in myocardial tissue and blood vessels.

Supplement: Figure S1. Expression of APJ in myocardial tissue and blood vessels. Representative images of immunohistochemistry for APJ in heart sections from C57 and diabetic mice as quantified in (B) (n=6 mice per group, *p< 0.05). Scale bars represent 50μm. [file supplementary_figure_1.pdf]

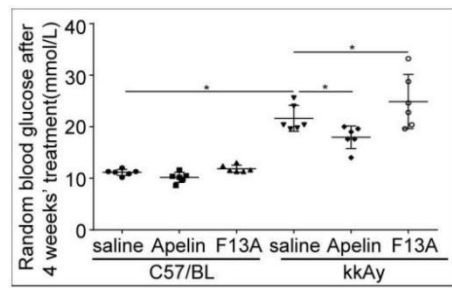

**Figure S2.** Random blood glucose after 4 weeks' treatment in C57 and diabetic mice.

Supplement: Figure S2. Random blood glucose after 4 weeks’ treatment in C57 and diabetic mice. Representative random blood glucose from C57 and diabetic mice with or without apelin/F13A treatment as quantified using unpaired Student’s t-test (n=6 mice per group, *p< 0.05). [file supplementary_figure_2.pdf]

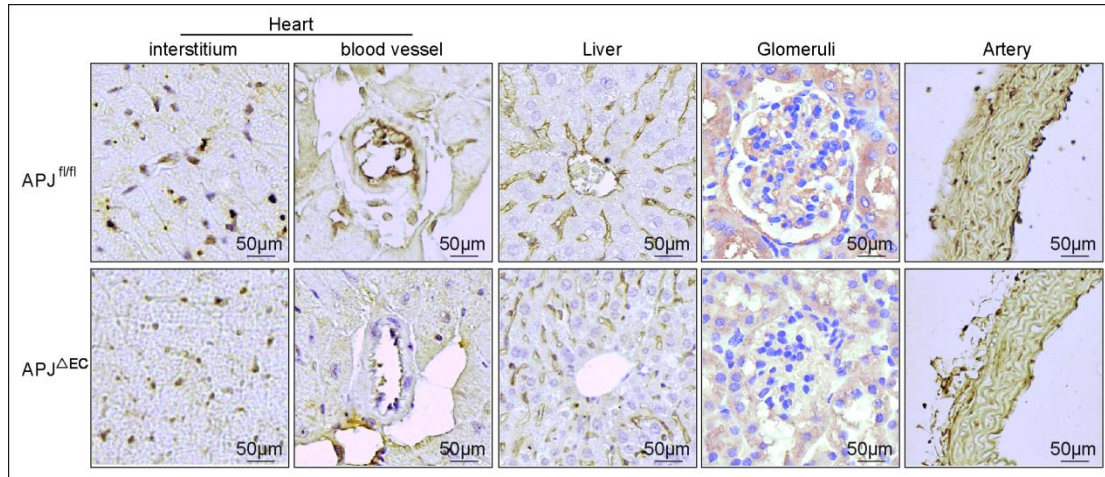

**Figure S3.** Expression of APJ in APJ<sup>fl/fl</sup> and APJ<sup>ΔEC</sup> mice.

Supplement: Figure S3. Expression of APJ in APJfl/fl and APJ△EC mice. Representative images of immunohistochemistry for APJ in heart, liver, glomeruli and artery sections from APJfl/fl and APJ△EC mice. Scale bars represent 50μm. [file supplementary_figure_3.pdf]

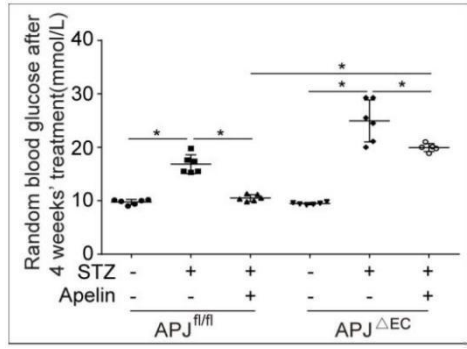

**Figure S4.** Random blood glucose after 4 weeks' treatment in  $APJ^{fl/fl}$  and  $APJ^{\Delta EC}$  mice.

Supplement: Figure S4. Random blood glucose after 4 weeks’ treatment in APJfl/fl and APJ△EC mice. Representative random blood glucose from APJfl/fl and APJ△EC mice with or without apelin treatment as quantified using unpaired Student’s t-test (n = 6 mice per group, *p< 0.05). [file supplementary_figure_4.pdf]
